# Maintaining Our Commitment to Building Scientific Publishing Capacity of Students: PCD’s 2022 Student Paper Contest Winners and Release of 2023 Call for Papers

**DOI:** 10.5888/pcd19.220303

**Published:** 2022-11-17

**Authors:** Leonard Jack

**Affiliations:** 1 Preventing Chronic Disease, Office of Medicine and Science, National Center for Chronic Disease Prevention and Health Promotion, Centers for Disease Control and Prevention, Atlanta, Georgia

*Preventing Chronic Disease* (PCD) initiated its first Student Paper Contest in 2011 (1). Since that time, the journal has received hundreds of student research papers from around the world. Last year, PCD celebrated its 10-year anniversary of building scientific publishing skills and abilities among students (2). Research mentors have used this journal as an avenue to introduce their students to the rigors of generating scholarly writings that focus on conceptualizing research; collecting, analyzing, and reporting data; and discussing the public health implications of research findings. Since 2011, regardless of whether papers are accepted, PCD has provided students with extensive feedback on their submissions.

## Goals and Submission Requirements

Eligibility for PCD’s student paper contest has evolved over the years (2). Participants must be currently enrolled students or have completed one of the following programs within the last 12 months: high school, undergraduate or graduate degrees, medical residency, or a postdoctoral fellowship conducted under the supervision of a principal investigator or research advisor. PCD also requires that the student author serve as the first and corresponding author. PCD only accepts original research that has not been published previously or submitted elsewhere for publication. Papers submitted for consideration must use one of two PCD article types: Original Research or GIS Snapshots.

Over the years, PCD has refined the purpose of the student research publication opportunity to include these 5 primary goals (3):

- Provide students with an opportunity to become familiar with a journal’s manuscript submission requirements and peer-review process
- Assist students in connecting their knowledge and training on conducting quality research with a journal’s publication expectations
- Develop students’ research and scientific writing skills to become producers of knowledge rather than just consumers of knowledge
- Provide students with an opportunity to become a first author on a peer-reviewed article
- Promote supportive, respectful, and mutually beneficial student–mentor relationships that strengthen students’ ability to generate and submit scholarly manuscripts throughout their professional careers

## 2022 Winners and Submissions

Sixteen student research papers were submitted for the contest in 2022, and all submissions underwent the same peer-review process as any other manuscript submitted to the journal for consideration. Eight of the 16 papers were successful in making it through a rigorous review process before being accepted for publication (4–11). Student research papers addressed a range of topics: COVID-19 and various aspects of health, food insecurities among caregivers in southern states, the relationship between physical activity and depression among high school students, perceptions of neighborhood development on active living among community residents, use of a cancer index as a predictor of common cancers, and spatial analysis of breast cancer mortality rates in rural states. PCD is pleased to announce 2022 student paper winners in 4 categories: high school, undergraduate, master’s degree, and doctoral degree. After careful review, PCD did not select a winner in the postdoctoral category.

In the high school category, Wang and coauthors of the article “Association Between Physical Activity and Sedentary Behavior With Depressive Symptoms Among US High School Students, 2019” point out that depression among high school students has increased over the past decade (4). Their research analyzed 2019 data from the Youth Risk Behavior Survey that consisted of 13,526 high school students. They found that increased exposure to computer or device use was associated with higher odds of inadequate physical activity and excessive sedentary behavior and depressive symptoms. Their findings suggest the importance of public health interventions that focus on implementing physical activity to reduce risk of depression among high school students.

This year’s winning paper in the undergraduate category was generated by Creech et al: “Physical Activity Among Adults in Rural Western North Carolina During the COVID-19 Pandemic” (5). This article examines the locations, reasons, benefits, and barriers to engaging in physical activity among adults living in rural communities during the COVID-19 pandemic. Using nonparametric measures, authors identified that, among a sample of 297 adults, most engaged in physical activity at home, in parks, and in neighborhoods. Primary reasons for participating in physical activity included getting out of the house, maintaining mental health, and engaging in some form of physical activity.

Schulz and colleagues’ article, “Spatial Analysis of Breast Cancer Mortality Rates in a Rural State,” is the winner in the master’s degree category (6). This article reports findings from a study in South Dakota that assessed what sociodemographic factors contribute to mortality rates and, using spatial analysis, explored how counties’ observed age-adjusted mortality rates compared with expected rates. A linear regression model was used to identify sociodemographic factors associated with breast cancer mortality rates and to compute new standardized incidence ratios (SIRs), after controlling for significant factors affecting mortality. Findings indicated that educational level and breast cancer incidence rates were significant factors associated with breast cancer mortality rates at the county level. Authors also used the SIR model to show the spatial distribution of mortality rates by county.

Seto and coauthors of the article “Differences in COVID-19 Hospitalizations by Self-Reported Race and Ethnicity in a Hospital in Honolulu, Hawaii” were selected as winners in the doctoral category (7). Pointing out that COVID-19 has exacted a tremendous toll on racial and ethnic groups in the US, they sought to identify the extent to which race and ethnicity were misclassified in COVID-19 hospitalizations. They assessed the responses of 847 patients at randomly selected hospital and ambulatory units who completed a survey that asked them to self-identify their race and ethnicity and compared their responses with data in electronic medical records (EMRs). Authors found that using self-identified data on race and ethnicity rather than hospital EMR data may help uncover further disparities in COVID-19 hospitalizations.

PCD congratulates this year’s impressive winners. In addition, we invite you to join us in celebrating all student authors who submitted manuscripts to the contest, regardless of whether their manuscripts were accepted. We hope all student authors who submitted papers gained a tremendous amount of experience serving as first and corresponding authors and gained more knowledge of journal submission guidelines and in the correspondence process with the journal’s editor in chief, peer reviewers, associate editors, and technical editors.

In closing, PCD is proud to release its 2023 Student Paper Contest call for papers. PCD is interested in student research papers relevant to the prevention, screening, surveillance, or population-based intervention of chronic diseases, including but not limited to arthritis, asthma, cancer, depression, diabetes, obesity, and cardiovascular disease. PCD is also interested in papers in these areas that explore the role of social determinants of health to include the impact of racism in shaping health outcomes. Readers are asked to encourage students at the high school, undergraduate, master’s, and doctoral levels, as well as medical residents and postdoctoral fellows, to submit a student research paper to the journal for consideration. Students are encouraged to submit an inquiry (www.cdc.gov/pcd/for_authors/submit_inquiry.htm) to the journal before the submission deadline to determine whether a given topic area and research focus align with the intent of the contest. For more information about the journal and previous collections of student papers, please visit the PCD website at www.cdc.gov/pcd.

## References

[1] Posner SF . PCD’s first annual student research contest: Lui and Wallace examine hospitalization rates for at-risk populations. Prev Chronic Dis 2011;8(5):A103. 21843406 PMC3181176

[2] Jack L Jr . PCD 2021 student research collection: building public health research capacity in real-world settings and the 2022 call for papers. Prev Chronic Dis 2021;18:E68. 10.5888/pcd18.210214 34237244 PMC8269742

[3] Jack L Jr . Shaping future generations of public health researchers: Preventing Chronic Disease’s student research paper contest. Prev Chronic Dis 2017;14:E96. 10.5888/pcd14.170431 29023229 PMC5645190

[4] Wang C , Peiper N . Association between physical activity and sedentary behavior with depressive symptoms among US high school students, 2019. Prev Chronic Dis 2022;19:E76. 10.5888/pcd19.220003 36395000 PMC9673959

[5] Creech W , Towner B , Battista R . Physical activity among adults in rural western North Carolina during the COVID-19 pandemic. Prev Chronic Dis 2022;19:E74. 10.5888/pcd19.220112 36395002 PMC9673977

[6] Schulz M , Spors E , Bates K , Michael S . Spatial analysis of breast cancer mortality rates in a rural state. Prev Chronic Dis 2022;19:E65. 10.5888/pcd19.220113 36265079 PMC9616131

[7] Seto B , Nishizaki L , Akaka G , Kimura J , Seto T . Differences in COVID-19 hospitalizations by self-reported race and ethnicity in a hospital in Honolulu, Hawaii. Prev Chronic Dis 2022;19:E72. 10.5888/pcd19.220114 36395004 PMC9673976

[8] Goswami S , Korgaonkar S , Bhattacharya K , Rosenthal M . Food insecurity in a sample of informal caregivers in 4 southern US states. Prev Chronic Dis 2022;19:E51. 10.5888/pcd19.220069 35980833 PMC9390796

[9] Tsuzaki K , Taira D . Forgone health care for non–COVID-19–related needs among Medicare beneficiaries during the COVID-19 pandemic, summer 2020–winter 2021. Prev Chronic Dis 2022;19:E64. 10.5888/pcd19.220110 . 36227851 PMC9616132

[10] Dsouza N , Serrano N , Watson KB , McMahon J , Devlin HM , Lemon SC , Exploring residents’ perceptions of neighborhood development and revitalization for active living opportunities. Prev Chronic Dis 2022;19:E56. 10.5888/pcd19.220033 36048735 PMC9480840

[11] Guo L , Wright M , Osias M , Vaezi M , Hughes M . Creation and evaluation of the Illinois cancer risk index as a predictor of four common cancers. Prev Chronic Dis 2022;19:E75. 10.5888/pcd19.220104 36395001 PMC9673961

